# Toward Optimal Learning of the Gesture in Laparoscopic Surgery: Methodology and Performance

**DOI:** 10.3390/jcm11051398

**Published:** 2022-03-03

**Authors:** Marine Cau, Juan Sandoval, Amaël Arguel, Cyril Breque, Nathalie Huet, Jerome Cau, Med Amine Laribi

**Affiliations:** 1 CLLE, CNRS, UMR5263, University of Toulouse, 31058 Toulouse, France; marine.cau1@etu.univ-tlse2.fr (M.C.); nathalie.huet@univ-tlse2.fr (N.H.); 2 Department GMSC, Pprime Institute, CNRS—University of Poitiers—ENSMA, UPR 3346, 86073 Poitiers, France; juan.sandoval@univ-poitiers.fr (J.S.); cyril.breque@univ-poitiers.fr (C.B.); 3 Groupe GIE Sainte Marguerite, Vascular Laparoscopic Surgery, 71 Boulevard Robespierre, 83000 Toulon, France; cau.jerome@gmail.com

**Keywords:** surgical gesture learning, laparoscopy, motion-capture, augmented reality, epistemic emotions, confusion, self-efficacy, feedbacks, disruptive environment

## Abstract

Classical surgical education has to face both a forensic reality and a technical issue: to train a learner in more complex techniques in an increasingly short time. Moreover, surgical training is still based on an empirical hierarchical relationship in which learners must reproduce a sequence of actions in a situation of strong emotional pressure. However, the effectiveness of learning and its quality are linked to the emotional states in which learners find themselves. Among these emotions, epistemic confusion can be found that arises in complex learning situations where there is a cognitive imbalance related to the comprehension of the task, and which results from a rupture between the pre-established patterns of the learner and the new learning task. Although one knows that confusion can have a beneficial or a negative impact on learning, depending on whether it is well regulated or not, the factors that can influence it positively are still poorly understood. Thus, the objective of this experiment is to assess the impact of confusion on the learning of a surgical procedure in an augmented reality context and to determine if this impact varies according to the feedback given to the learners and according to the occurrence of disruptive events. Medical externs were recruited (*N* = 15) who were required to perform a suturing task on a simulator and whose performance was measured using a Motion Capture (MoCap) system. Even though the statistical analyzes did not allow a conclusion to be reached, the protocol already established makes it possible to consider a longer-term study that will allow (by increasing the number of sessions and the number of participants) more significant results to be obtained in order to develop new surgical learning protocols. This preliminary study opens a new field of research on the influence of epistemic emotions, and more particularly of confusion, which is likely to upset traditional surgical teaching, and is based on negative conditioning and strong emotions with negative valence as well as stress and coercion.

## 1. Introduction

The development of new complex surgical techniques such as laparoscopic surgery and robot-assisted surgery is more demanding for trainees and requires specific training [1,2,3]. This training, based mainly on simulators, is becoming mandatory to acquire the necessary skills, and to gain security for the patients. Thus, the recent development of new technologies applied to medicine, such as augmented reality, opens up new perspectives on the very nature of surgical learning via those interfaces [2]. However, if optimizing the acquisition of these technical skills would reduce the learning curve [1], education on simulation does not take into account all the components necessary to react in the context of an operation. Indeed, if an unexpected event or a complication occurs during surgery, the correct decision making is mandatory for the patient’s life. If the training on a simulator is fit to acquire new skills for the trainees, it does not allow learning in real situations.

Furthermore, this major issue cannot be solved by simple technologies, which is why our multidisciplinary team (robotic engineers, surgeons, and psychology researchers) decided to study how to improve this training by the association of psychological learning (e.g., emotional control, flexibility, etc.). Indeed, emotions appear to be closely linked to learning in academic learning, or, more generally, in complex learning situations. Indeed, the effectiveness of a learning activity, as well as its quality, is linked to the emotional states that learners can experience [4,5,6].

In our study, the epistemic emotions that arise during a complex learning situation were considered. Among these emotions, notably, we considered epistemic confusion, which arises in complex learning situations when there is a cognitive imbalance hindering understanding and resulting from a rupture between pre-established patterns of the learner and new information brought by the learning task [7,8,9]. Although one knows that confusion can have a beneficial or negative role in learning, depending on whether it is well regulated or not, the factors that can influence it positively are still poorly understood [10,11,12,13]. Therefore, objectifying and understanding the occurrence of confusion, as well as the factors that allow it to be well regulated in order to make it optimal (such as feedback or learner’s self-efficacy belief), will allow more effective learning towards a resolution of the problem, and a higher engagement in the task that the learner must achieve. Hence, this experimental setup based on motion capture system (MoCap), developed by the CNRS, has enabled us to set up specific teaching of complex gestures on a simulator retro-evaluated in real time by a computer.

The contribution of this research is both to objectify the influence of unexpected events on the fluctuation of epistemic emotions in gesture learning in pre-real condition of a surgery (with augmented reality) and to consider the possibility of a positive resolution of emotional states, which are negative to optimal learning, through psychological training adapted to such events (self-efficacy belief). Understanding this phenomenon would make it possible to establish a teaching method so that the learner can quickly cope with the real conditions of an operating room, which require cognitive adaptation, as well as an ability to quickly solve problems in front of a disruptive environment.

## 2. Surgery Evaluation Criteria

### 2.1. Gesture Learning

Learning the surgical procedure, prior to the operating room immersion, seems obvious. Optimizing the acquisition of these technical skills would reduce the students’ learning curve [1,2,3]. Indeed, the development of modern laparoscopy techniques requires special training in order to face the difficulty of this gesture mastering. The two main difficulties of this technique are the loss of 3D vision and the depth of the instruments’ movements [1].

New learning techniques are then developed, they are based on theories that establish scales for the acquisition of motor skills and scales for the development of overall surgical expertise. This unique training is based more particularly on the theory of Fitts and Posner [1,2,3,14], which covers the three stages of motor skills acquisition (Objective Structured Assessment of Technical Skills (OSATS)) in which candidates perform a series of standardized surgical tasks on inanimate models under the direct observation of an expert. This Fitts and Posner model has been reviewed by the CNRS: this original four-step training method, tested in their research report on motion analysis in minimally invasive surgery, can be used for all types of training. This training is carried out in four stages, divided as follows:

1. Cognitive stage: The first stage is the acquisition of the basic gestural technique.
2. Contextual stage: The second stage is the acquisition of basic or advanced techniques in a situation, on a mechanical or animal model, by specialty. The basic gestures being acquired, it is a question of now restoring this gesture in its environment. Each manipulation is evaluated by a specific score in order to develop a performance score. The performance score includes the gesture scores (measured by the MoCap system) and a suture one (evaluated on a specific score established by the vascular laparoscopic expert).
3. Operational stage: The third stage is the acquisition of operative surgical strategies, by the transmission of traps and operative solutions.
4. Feedback stage: The fourth stage is the transmission of the acquired knowledge.

In order to decrease the learning curve and make it more efficient, the contextual and operational stages of the model were grouped and, thus, combined the acquisition of techniques in situation and the acquisition of surgical strategies by the transmission of traps and operating solutions. In fact, if the fusion of the contextual and the operational stage is innovative and new in the surgery field, it is based on an existing model of everyday life that has shown its effectiveness: video games [15]. That is why new questions arise about gesture learning with the development of innovative technologies, such as mixed reality.

### 2.2. Augmented Reality and Surgical Learning

The quick development of technologies allows the advancement and integration of their benefits in higher education. Some authors have determined that an augmented reality environment would have a positive impact on learning, and more specifically on learner engagement with, and motivation for, the task [16,17,18]. However, most of the actual research studies do not define exactly which factors are able to positively influence students’ motivation and, more generally, the effects and implications of augmented reality in education [19]. There is consensus that technology should be primarily used to analyze data or solve a particular problem [20]. For Dewey [21], learners must participate actively in learning, in order to relate the current content offered by the task to previous experiences and knowledge already acquired. Augmented reality could allow this association and therefore facilitate engagement in the task [16,17,18].

The challenges of surgical training are therefore to reduce training time and optimize the acquisition of skills without taking any risks for the patient [1,2,3]. The only two levers in order to meet these two requirements, and thus reduce the learning curve, are firstly the acquisition of the gesture before being in the operating room situation and, secondly, to develop new tools in order to make the intervention more efficient. To avoid errors and negative learning outcomes in surgery (and more particularly in laparoscopic surgery in this study), proper training is of the utmost importance. Simulation is a safe way to train surgeons in laparoscopic skills. For this purpose, traditional simulators are used, but they lack objective performance evaluation [22].

## 3. Emotions and Surgical Learning

### 3.1. Epistemic Emotions and Confusion

Emotions are linked to learning, whether in academia or in complex learning situations [1,2,3]. According to the control value of accomplishment emotions theory [23], learning has an impact on the emotions of students, through their cognitive evaluation. Pekrun investigated the link between emotion and motivation and he stated that it is a determining factor in learning, and therefore in students’ academic performance. When emotions are activated, they can influence performance through cognition, motivation, and accomplishment behavior [22]. That is particularly relevant with epistemic emotions, which are affective states occurring along the cognitive processes triggered by complex tasks such as learning. For instance, the state of confusion can be beneficial for learning when it leads learners to invest more effort in activities in order to reduce this negative emotion. Considering epistemic emotions such as confusion can hence be useful for medical education. A theory has been developed specifically to address learning and performance tasks resulting in pass or fail results [24,25].

Confusion is frequent in complex learning situations, for example when a piece of information is not consistent with some student’s prior knowledge. This creates a break in the prior cognitive schema of the student who is facing the task. According to Mayer [10], complex learning requires the simultaneous processing of new information while retrieving prior knowledge in memory, and ultimately integrating these two pieces of information together into a new mental model. When a step of learning is not correctly processed, or if it is interrupted, it causes cognitive disequilibrium resulting in a state of confusion. However, confusion can be beneficial for learning when it promotes a deeper engagement, which can lead to better understanding and positive experience with the task [12]. To illustrate, an optimal zone of confusion [7,12] can be considered. When a student is located within this zone, they are able to put in place strategies that will allow them to solve the problem they are facing. If they fail, they consequently enter a so-called suboptimal zone of confusion, and then a state of frustration. If frustration persists, it will eventually lead to negative outcomes, such a disengagement from the task [12,13].

The relationship between individual variables (including prior knowledge, self-efficacy, and self-regulation), the structure and design of tasks during learning, and finally the form of feedback and support given to students, determine the degree of difficulty experienced by the learner, as well as the impact of emotions (productive or unproductive) on learning performance.

### 3.2. Self-Efficacy

Self-efficacy is the belief that an individual has of their ability to succeed in a task [26]. If this belief is positive and strong enough, it can lead to making more efficient decisions in difficult situations. A person who possesses a strong self-efficacy belief can attribute failure to insufficient effort [27]. People deal with threats or stressors in the belief that they can exercise some control over them. This perspective of effective mastery and control improves performance and reduces stress [27]. In his work, Bandura [28] starts from the hypothesis that self-efficacy expectations make it possible to determine whether an adaptive behavior will be produced, how much effort will be expended, and for how long it will be sustained when difficulties and aversive experiences occur. In other words, cognitive, emotional processes and the influence of the social environment alter the level and strength of self-efficacy belief.

One of the most important sources of information that can induce self-efficacy beliefs, persuasion by others, is of particular interest to us. It makes it possible to positively influence a learner’s self-efficacy belief by showing them, beforehand, support and improving self-confidence in their ability to adapt themselves to difficulties (for example, to perform a suturing task), and in their abilities to perform a task, through verbal suggestions [28,29].

Self-efficacy belief was investigated in order to determine whether it can produce a positive impact on the management of epistemic confusion, hence contributing to participant’s engagement in the learning task.

## 4. Materials and Methods

### 4.1. Population

We recruited surgical externs (*N* = 15), by using a Google Form advertisement from the Faculty of Medicine and from the Poitiers University Hospital, France. Surgical externs were 5th-year medical students, also called in UK Senior House Officer (SHO). There were 6 women and 9 men aged between 22 and 25. The participants had never before performed suturing tasks in laparoscopy context.

#### 4.1.1. Experimental Design

The experimental design is a factorial design with independent measures. There are 3 independent groups defined by the intersection of the 2 independent variables (whose description can be found in Table 1: self-efficacy instruction (with or without) and the instance of unexpected events via stimulation through the HoloLens-2 headset (virtual stimulation vs. no stimulation).

Participants were randomly assigned to three different groups, using randomization software. These groups were the control group or CG, and the two experimental groups. The first experimental group (*n* = 6), called “No Self-efficacy Belief” (NSEB), did not benefit from the instructions favoring self-efficacy; they were equipped with the HoloLens-2 helmet. The second one (*n* = 5), called “Self-Efficacy Belief” (SEB), benefited from the instruction favoring the feeling of self-efficacy and had to perform the task with the HoloLens headset. Finally, the control group (CG) (*n* = 4) had no instruction favoring the self-efficacy belief nor the HoloLens-2 headset.

#### 4.1.2. Hypothesis

The main purpose of this research is to identify the influence of unexpected events on the dynamics of emotions during the gesture learning in a situation of an operating theaters simulation (augmented reality) and then consider the possibility of managing negative learning emotional states by prior intervention adapted to such events (via an instruction to increase the self-efficacy belief). The aim is to understand the effect of the mismatch between prior knowledge patterns and feedback (congruent or discordant) on the triggering of epistemic emotions (confusion) as new information is acquired and the ability to react quickly to incongruent or disruptive events.

Thus, it is expected that confounding status will vary significantly between groups. A statistically significant difference between groups SEB and NSEB in terms of level of confusion is expected. Specifically, the SEB group that received the verbal persuasion instruction toward increased self-efficacy was expected to have a more optimal level of confusion than the NSEB group without the instruction. This could be explained by the fact that the verbal persuasion instruction provides a baseline of the participants’ ability to solve the difficulty in the task at hand. Whereas for the group without the verbal persuasion instruction, the participant is expected to exhibit a more persistent state of confusion that tends toward suboptimal confusion.

Moreover, a statistically significant difference between groups SEB and NSEB in terms of hand movement and quality of the suture is expected. Indeed, as a result, the group with verbal persuasion was expected to show faster and more efficient learning of the laparoscopic suturing gesture than the group without the initial instruction.

### 4.2. Protocol

The protocol is divided into two distinct phases. The first was the pre-test phase during which the participants were able to benefit from an initial approach to the task. An expert shows the participants how to perform the suturing task that they will have to perform during the test phase. The participants were able to train in mastering the complex gesture of anastomosis-end-to-end suture, and thus familiarize themselves with the gripper and the needle holder to avoid non-habituation bias that could impact performance.

The second phase was the test phase based on the Fitts and Posner model of acquisition. The contextual stage corresponds to the acquisition of basic/advanced techniques on a mechanical model (here is the learning of the gesture and the suture skills on the pelvis-trainer). The operational stage is the acquisition of surgical strategies by the transmission of traps and operational solutions. This stage was integrated by the restitution of disruptive events and feedback given by the HoloLens headset. When participants arrived in the room adjoining the test room, they were invited to read the study departure instructions aloud. After reading these instructions, the participants were required to answer a written question to measure the feeling of self-efficacy: “Indicate your degree of confidence in your ability to perform the task with precision by indicating the corresponding percentage in the scale below: from 0%—completely uncertain to 100%—quite certain.” Then, before each session, the MES was given to the participants in order to measure their emotions before the task. The participants from all conditions (SEB, NSEB, and CG) were then invited to take place in the examination room in order to perform the suturing task. The CG was required to simply perform the task without distractors or feedback during the task. Participants in the SEB and NSEB groups were equipped with the Microsoft HoloLens-2 augmented reality headset by the lab team. Participants of all conditions were required to stand in front of the suturing simulator (pelvic-trainer), picking up their instruments, and completing the suturing task within 20 min. The HoloLens-2 headset delivered pre-recorded audio and feedback to the participants. The instruments (clamp and needle holder) held by the participants were equipped with small markers, detected by the cameras in the room, in order to observe and measure the quality and efficiency in the realization of the gesture via the capture of movement system (MoCap). The score was then compared with an expert gesture score. In order to see whether our hypothesis on the performance of gesture learning in a disruptive environment holds or not, the study took place over three individual sessions of 30 min each, over a fairly short time interval (2 weeks). The participants were asked to complete the self-reported questionnaire, the Medical Emotion Scale (MES) [30], before and after each session [29,31].

### 4.3. Work Environment

#### 4.3.1. Self-Efficacy Instructions

Two instructions were drawn up: the first instruction consisted of an explanation regarding the course of the session and a summary of the historical development of laparoscopy used for the control and NSEB groups, and the second instruction was an explanation regarding the course of the study associated with a verbal persuasion instruction for the SEB group in order to give the participants enough confidence in their ability to succeed in the task and, thus, to reinforce personal efficiency by diverting attention from negative thoughts [29]. The persuasion instruction given to the SEB group was developed by the authors, based on pre-established templates that were modified to suit our study. The self-efficacy information was given to the participants in paper format (A4 sheet). Participants were required to read out loud the instruction that consisted of a short text aiming at improving self-efficacy. The text basically reminded them that difficulties are normal during learning, that they can handle the task, and that they must stay focused on what they are doing (Appendix A).

#### 4.3.2. Motion Capture System Setup

**Figure 1:**
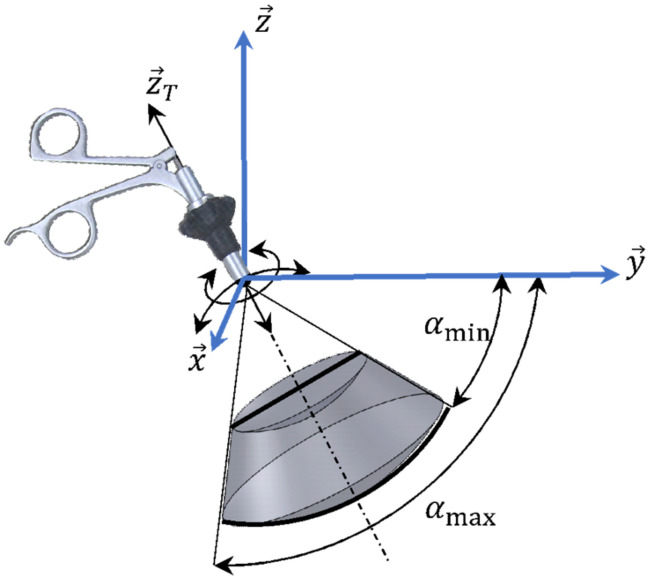
Orientation angle description.

A Motion Capture system (MoCap) was used to measure the surgeon’s gestures during the task execution by computing the orientation angle of each surgical instrument, as shown in Figure 1. These angles, defined as physical description, allow evaluating the workspace size within each instrument moves as well as the motion’s quality during the task [2,3]. Previous studies have been conducted with experts, which identified the mean value of the apex angle, which is equal to 52°. Since the tools always have to go through the incision point, the workspace size is evaluated through the calculation of the measured angle of the cone swept by the axis of the instrument tool. This cone is described by an apex angle. This value represents the maximum value recorded on all manipulations performed by 10 surgeons in a MIS environment [1,32].

Thereby, the smaller the workspace is, the more effective and more focused the surgeon’s suture actions on the maneuver will be [33]. In order to evaluate the suture efficiency, a scoring method was defined by a vascular laparoscopic surgery expert [34,35]. In the same way as an efficient suture on patients is computed, the score was based on regularity and thickness criteria with regard to the quality of the suture, the number of leaks, and the distance sutured. Moreover, a gesture score was established on the average results by four laparoscopic surgeons, for end-to-end vascular suture, performed in the same condition with the MoCap system, in order to measure task performance.

The movement of the participants were recorded in the laboratory using equipment dedicated to learning laparoscopic techniques: the Pelvic-trainer. Two instruments were used by the subjects, a forceps (AESCULAP^®^ P0841SU, single use) and a needle holder (MICROFRANCE^®^). The needle holder automatically maintains the needle clamping force without maintaining finger pressure. The clamp does not allow this maintenance. The needle holder is grasped by the right hand and the gripper by the left hand. To follow the three-dimensional movement of the tools, sets of reflective passive markers are positioned on the needle holder, a first one in the axis of the shank (markers pierced by the shank), and a second one eccentric allowing its own rotation measurement. The gripper has two families of reflective markers as well, as the shank has a degree of free rotation around the handling. Markers are placed on the rod, one in the axis (markers pierced by the rod) and an eccentric to measure its own rotation. This rotation is controlled by the practitioner using a wheel.

**Figure 2:**
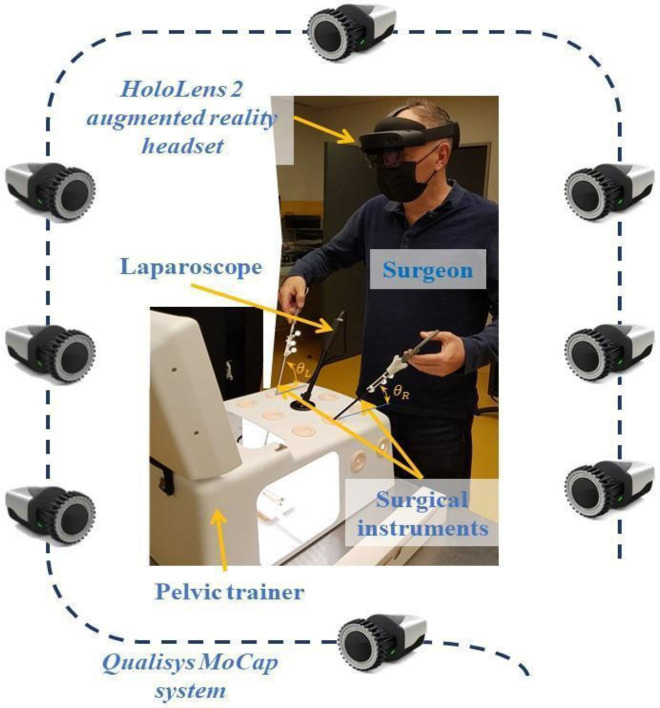
Experimental platform for gesture recording and evaluation.

The motion acquisition system consists of eight Miqus cameras from Qualisys^®^, synchronized via the Qualisys Track Manager system. This reports the gesture results, which are interpreted in terms of the angle of rotation, with a score for the gripper and a score for the needle holder, respectively. The results are then compared to those of the expert. The eight cameras were placed in front of the subject, avoiding being obstructed by the Pelvic-trainer’s screen. The operating table is adjustable in height and positioned in front of the force platform (Figure 2). A video camera was attached to the pelvic-trainer to record the task performed inside it (distance between the ends of the tools: approximately 20 cm). An 8/20 mm poly-mesh Perouse^®^ vascular prosthesis was used (knitted polyester vascular grafts, indicated for the replacement or bypass of arteries), the suture task had to be performed over 15 cm, with a Surgipro^®^ 4/0 thread lengthwise useful 15 cm, whose needle was 22 mm 3/8th.

#### 4.3.3. Self-Reported Questionnaire (Emotional State)

In order to measure the state of confusion of participants and thus observe the effect of the self-efficacy belief on the state of confusion, a self-assessment questionnaire was used, the Medical Emotion Scale [30]. In medical education, MES can be used to assess the intensity of emotions in order to better understand the impacts of these emotions on learning and performance in medicine. The MES is based on a five-point Likert scale (ranging from 1 = “not at all” to 5 = “very strongly”), which measures the intensity of emotions. This self-reported questionnaire allows the study of discrete emotions, and groups these emotions into more general affects according to their valence (pleasant or unpleasant) and their physiological activation level [30]. The scale consisted of 22 adjectives to measure basic, epistemic, and social emotions. The MES is also divided into four subscales created according to their valence: (1) positive activation (pleasure, pride, curiosity, joy, recognition, compassion, hope); (2) positive deactivation (relaxation, relief); (3) negative activation (anxiety, frustration, confusion, fear, anger, shame, disgust); and (4) negative deactivation of emotions (hopelessness, disappointment, sadness, boredom). In addition, the adjectives “neutral” and “surprise” were included as states of non-valence (neutral subscale). The test was presented in paper format (A4 sheet). The first test was given before the task, and the second one after the task, for all of the three examinations (Appendix B).

#### 4.3.4. HoloLens-2 Headset—Feedback and Disruptive Events

The HoloLens-2 headset was used to give feedback (congruent and incongruent with the task) to the participants [2]. These feedbacks were displayed as a hologram, in a black but not opaque square. In order not to disturb the participants, the team made the hologram stable with respect to the real world, i.e., it did not follow the movement of the participants’ heads but remained fixed above the pelvic-trainer screen. For this, audios and videos were directly downloaded into the headset, and played through the VLC player already implemented in the HoloLens 2 headset.

The video appeared for a few minutes per session (visual feedback from an expert performing the task for to the participants). Further, audios composed of different sounds were used. These audios had a feedback function and some of their elements had a disruptive function. Three audios were therefore created using audio editing software, each audio corresponded to a session (audio1-session1, audio2-session2, and audio3-session3) and all audios were standardized for all participants in the SAE and NSAE groups and made as neutral as possible. Each audio was 20 min long and indicated the beginning and end of the session to the participants (see Appendix C—detailed description of audios). The first audio provided simple feedback to participants, such as “focus” or “place your hands correctly”. A disruptive event was added to the second audio through the following lecture question: “Name out loud five collateral branches of the subdiaphragmatic abdominal aorta”. Finally, the third and last audio was punctuated by random sound “beeps”, disrupting the participants’ task, and without additional indications, forcing them to question themselves, or not, and to correct, or not, their gesture.

### 4.4. Statistical Tests Applied

The results were expressed as means, medians, and standard deviations for the quantitative variables (performance in learning the gesture and emotional state or more precisely the level of confusion felt). A simple descriptive analysis was performed on the whole study population and then by subgroups. This description focusses on the MoCap data expressed in instrument rotation angle as well as the suture performance (the combination of the two creates the variable “gesture learning”), the level of felt confusion state, the valence of this state (optimal vs. suboptimal). The parameters of position were computed (mean, median, min, and max obtained and the first and third quartiles given for the series, which separates the lower 25% present in the data from the upper 25%). We checked the existence of outliers and the normality of the residuals (Shapiro-Wilk test). The homoscedasticity was tested (Breusch-Pagan Student test) as was the independence of the residues (Durbin-Watson test).

The confidence interval of the mean was 95%. The standard deviation, the variance, the interquartile range were used as parameters to characterize the dispersion of the data. The Kolmogorov-Smirnov normality test was used for each of the variables of interest to verify if the data follow the normal distribution. In order to test the impact of independent variables on performance, a Kruskal–Wallis test was performed. Then, a relationship between the feeling of self-efficacy and the level of confusion and between the level of confusion and the learning performance of the gesture were investigated, for which the confidence interval was 95%, using post hoc tests (Dwass-Steel-Critchlow-Fligner pairwise comparisons).

## 5. Results

### 5.1. Descriptive Results

Descriptive statistics for the emotional state (whether it is positive or negative and before and after the task) and for the self-efficacy belief are provided in Table 2. Fifteen participants (*N* = 15) were included in the study. The mean score for self-efficacy was 39.7 (M = 39.7, SD = 19.1). Emotions were classified into activation level subscales, descriptive statistics for these variables are provided in Table 3.

Regarding the performance scores, represented by the suture scores (*M* = 10, *SD* = 2.33) and the gesture scores for the clamp and the needle holder (clamp: *Mdn* = 50.2, *SD* = 3.45; needle holder: *Mdn* = 51.5, *SD* = 12.4), the participants seem far from the score provided by the expert for comparison (clamp: *Mdn* = 41.8; needle holder: *Mdn* = 44.7). The means, the standard deviations, and the median of the dependent variables (emotional state and performance) and by experimental group are available in Table 4.

The normality of the sample was not good. The homoscedasticity of the residuals was greater than *p* > 0.5. The independence of the residues was greater than *p* > 0.5. We can therefore conclude that there is no doubt about the quality of the analyzes.

### 5.2. Inferential Results

The results of the non-parametric test show that there are no statistically significant differences for the median of the variable ‘self-efficacy belief’ between the CG and the NSEB and SEB groups (H (12) = 8, *p* = 0.1). The emotional state was measured before and after the task. In addition, the emotions were grouped into activation level: (1) positive activation (pleasure, pride, curiosity, joy, recognition, compassion, and hope) before the task (H (19) = 13, *p* = 0.1) and after the task (H (13) = 14, *p* = 0.6); (2) positive deactivation (relaxation, relief) before the task (H (8) = 5, *p* = 0.1) and after the task (H (4) = 5, *p* = 0.5); (3) negative activation (anxiety, frustration, confusion, fear, anger, shame, and disgust) before the task (H (35) = 30, *p* = 0.2) and after the task (H (12) = 9, *p* = 0.2); and (4) negative deactivation emotions (hopelessness, disappointment, sadness, and boredom) before the task (H (4) = 8, *p* = 0.8) and after the task (H (10) = 7, *p* = 0.2). However, the results indicate that there are no statistically significant differences between groups (CG, NSEB, and SEB) for the median of emotional state for all activation subscales either before or after the task.

There were no statistically significant differences between the CG, NSEB, and SEB groups concerning the gesture performance (suture scores and MoCap data) whether it was for the suture score (H (11) = 8, *p* = 0.2) or the gesture score (H (44) = 44, *p* = 0.5).

### 5.3. Motion Capture Results

**Figure 3:**
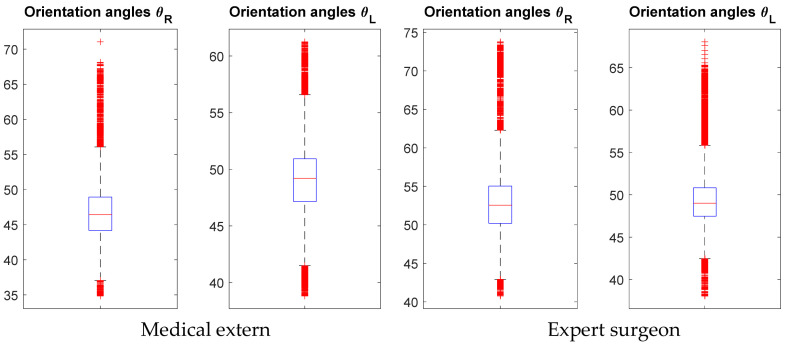
Orientation angles distribution for suturing task for Medical extern and Expert surgeon.

In order to show the effectiveness of using the motion capture and illustrate experimental results. Figure 3 shows the distribution of the orientation angles $\theta_{R}$ ∈ ℜ, for the clamp (right-handed), and $\theta_{L}$ ∈ ℜ, for the needle holder (left-handed). Measures of orientation angles (32,527 and 65,535 for an expert surgeon (ES) and SHO, respectively) were analyzed during the suturing task, mean $\theta_{R}$ was higher for ES (mean of 53.04°, interquartile range, 62.29–42.93°) than for ME (mean of 46.44°, interquartile range, 56.06–34.05°). While mean $\theta_{L}$ was lower for ES (mean of 49°, interquartile range, 55.83–42.42°) than for ME (mean of 49.19°, interquartile range, 56.61–41.49°).

**Figure 4:**
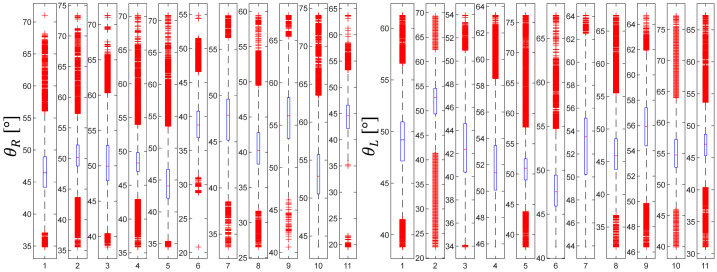
Orientation angle distribution for the first group.

**Figure 5:**
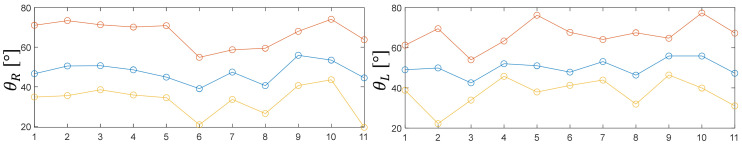
Evolutions of the maximum, median, and minimum values of the orientation angles for the first group.

**Figure 6:**
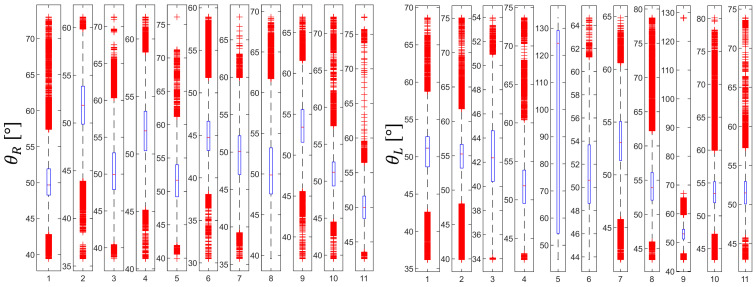
Orientation angle distribution for the second group.

**Figure 7:**
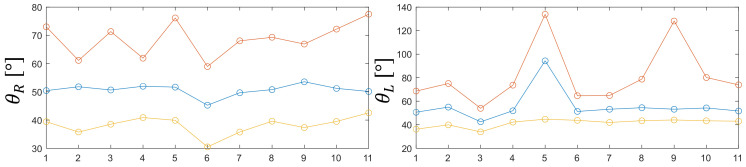
Evolutions of the maximum, median, and minimum values of the orientation angles for the second group.

**Figure 8:**
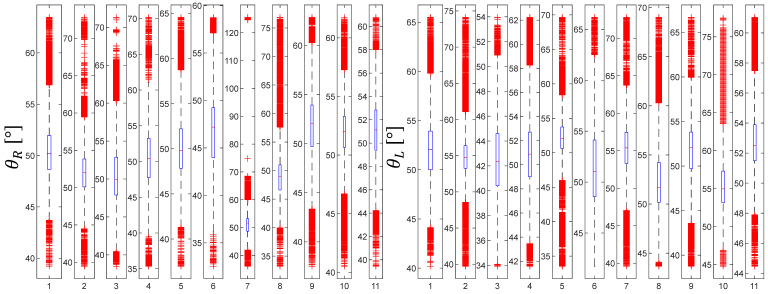
Orientation angle distribution for the third group.

**Figure 9:**
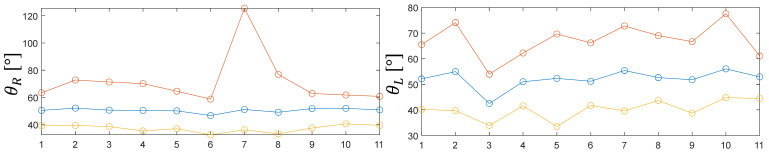
Evolutions of the maximum, median, and minimum values of the orientation angles for the third group.

Statistical analysis comparing the difference in $\theta_{R}$ and $\theta_{L}$ values between the groups is presented in Figure 4, Figure 5, Figure 6, Figure 7, Figure 8 and Figure 9. Evolutions of the maximum, median, as well as minimum values of the orientation angles, were highlighted inside each group.

Figure 5, Figure 7 and Figure 9 show the distribution of the orientation angles $\theta_{R}$ ∈ ℜ, for the clamp (right-handed), and $\theta_{L}$ ∈ ℜ, for the needle holder (left-handed) for the first, second, and third groups, respectively. Measures of orientation angles (65 535 for the three groups) were analyzed during the suturing task, mean $\theta_{R}$ was lower for the first group (mean of 50.07°, interquartile range, 37.55–66.6°) than for the two other groups (mean of 55.65° and of 52.07°, interquartile range, 41.48–81.4° and 40.18–67.15°, for the second and third group, respectively). While mean $\theta_{L}$ was in the vicinity for all the groups (mean of 48.95°, 50.62°, and 50.47; interquartile range, 33.08–66.86°, 38.14–68.76°, and 37–71.7°, for the first, second, and third group, respectively).

## 6. Discussion

The main results of the statistical analyzes of emotional state seem to indicate that the participants felt rather curious, grateful, and happy before and after the task. It also seemed that the participants felt rather frustrated and disappointed after the task. This can be explained by the difficulty of acquiring the suturing gesture under laparoscopy. This difficulty was not always correctly understood by the participants before the task. There was no significant difference in the emotional activation levels. Regarding performance, calculated on the basis of suture scores and data from the MoCap (forceps and needle holder), the results tend to show the difficulty for the participants to correctly manage the task. For the MoCap scores, the participants presented lower scores than those of experts (forceps: Mdn = 41.8; needle holder: Mdn = 44.7). This seems to point out that the movement was not performed in an optimal way, which included unnecessary gestures and rotations of the instrument. Similarly, the suture scores were quite high regardless of the groups (SEB: M = 9.4, SD = 2.3; NSEB: M = 10.1, SD = 2.3; CG: M = 10.9, SD = 2.3), the lowest score being 12, and the highest score being 3. A link cannot be established between immersive mixed reality learning and a more efficient and optimal learning when managing unforeseen/disruptive events, nor in the gesture mastery itself.

A larger sample seems necessary to verify the results obtained in this study in further research. Due to the current pandemic context, it was not possible to have a larger sample. In fact, medical students are currently being requisitioned as reinforcements in hospital services in order to provide labor during this period of the COVID-19 crisis. It seems important for us to specify that the protocol is cumbersome, complex, and subdivided into three sessions per participant, which requires great availability from the laboratory teams as well as from the participants.

The lack of significance of statistical tests may also be due to the subjective aspect of analyzing emotional state through self-reported questionnaires. Indeed, certain emotional states are difficult to identify consciously by those who feel them [36]. The confusion measure was mainly based on a single questionnaire item of the MES [30] and a more elaborate measure could have shown more conclusive results. Therefore, in future work, it would be appropriate to test the consistency of emotions between subjective (self-reported), behavioral, and physiological measures. Among the physiological measures, electrodermal activity can be included as its variations are measured during the performance of cognitive tasks [1,7]. In addition, heart rate variability is a good indicator of emotional variation and is a good physiological indicator [1,7]. Since confusion should trigger physiological changes, it should induce visible variations signals. These techniques can be considered as objective measures of confusion because they are the result of mostly unconscious and uncontrolled reactions of the autonomic nervous system [7,37,38,39,40]. It would also be interesting to vary the unexpected events and, thus, set up more intense medical events or tasks with higher stakes [30].

Fostering a learner’s self-efficacy belief will allow better regulation of the emotional state and, more particularly, of the state of confusion. The purpose of the verbal persuasion instruction was to encourage learners to develop self-regulation strategies in order to more easily cope with situations that can generate this state of confusion. Manipulated factors that can influence the occurrence of epistemic emotions, and particularly of confusion, seem to be a powerful method to improve learning experience in complex situations such as surgery. This approach is relatively new in the field of academic learning and professional training but represents a high potential and it calls for further research.

Self-regulation is the management of one’s own cognitive processes in pursuit of goals [41,42]. The results indicate that there are no significant differences between the three groups in the level of self-efficacy belief (i.e., their belief in their ability to organize and perform actions to achieve an expected result [28]) and the emotional state of the learners. The initial setpoint may not have been enough to create a high level of self-regulation. This is why it would be interesting to consider other ways to promote the self-efficacy belief. However, the effects of verbal persuasion can be only temporary if the learner’s efforts do not lead quickly to results [29]. In order to prevent this, learners can self-repeat the positive statements that relate to themselves. Repeating these statements enhances self-efficacy by diverting attention from negative thoughts or irrelevant feelings [28]. Objectifying and understanding the occurrence of confusion, as well as the factors that allow it to be well regulated in order to make it optimal (such as feedback or learner’s self-efficacy belief), will allow more effective learning, towards a resolution of the problem and a higher engagement in the task that the learner must achieve.

In our study the HoloLens-2 was only used to play pre-recorded audios and videos to the participants. Thanks to this technique, one can first observe which functions of the headset are useful in laparoscopic surgery learning situations, and thus be able, in a future study, to implement it with the necessary software. Since augmented reality is a combination of physical reality and virtual reality, it tends to improve physical training in laparoscopic simulation through the superposition of information or by an objective evaluation at the end of the performance. In order to prepare learners to deal with confusion, it seems relevant to give personalized advice that will indirectly encourage the participant or even increase the participants’ interest in the task. Thus, these interventions can have an impact on how learners experience confusion [12,43,44].

The results suggest that the variables explored do not have a significant impact on learning. However, these results should be interpreted with caution as this is an exploratory study, using an original method, and with a small sample, which could have reduced the statistical power of the analyzes. Thus, the exploratory nature of this research allows for future protocols to be considered in order to overcome this lack of significance.

## 7. Conclusions

The aim of this research is both to study the influence of unexpected events on the dynamics of emotions in the learning of a gesture in a situation of an operating theater in simulation (via mixed reality and the pelvitrainer) and to consider the possibility of managing the learnt negative emotional states by prior intervention adapted to such events (via a verbal persuasion instruction to increase the feeling of self-efficacy).

Although the results of the study do not validate the hypotheses, the exploratory nature of this research allows for future protocols to be considered. It would be interesting, as a first step, to reproduce the study with a larger sample in order to verify the results obtained in this study. It would also be interesting to replicate this study by including more accurate measures of emotional state (i.e., physiological and behavioral measures), personalized feedback, and more details and complete instructions promoting the self-efficacy feeling. This would make it possible to push research further towards the creation of new surgical training programs. To conclude, if future studies demonstrate their effectiveness in teaching complex techniques, one can imagine that such methods could be developed in all fields requiring such a level of success.

## Figures and Tables

**Table 1:** Description of the independent-measures factorial design.

		Virtual Simulation	(HoloLens-2)
		With	Without
Self-efficacy	With	CG	NSEB
instruction	Without	- -	SEB

“SEB” = Self-Efficacy Belief group; “NSEB” = No Self-Efficacy Belief group; “CG” = Control Group.

**Table 2:** Top five most felt emotions (positive/negative) before and after the task.

Time	Positive	*M* (*SD*)	Negative	*M* (*SD*)
Before	Curious	3.9 (1.0)	Anxious	1.8 (0.7)
	Grateful	3.7 (0.9)	Scare	1.5 (0.8)
	Happy	3.5 (1.0)	Surprised	1.3 (0.8)
	Optimistic	3.0 (0.9)	Confused	1.2 (0.5)
	Determined	2.9 (1.0)	Frustrated	1.2 (0.5)
After	Grateful	3.8 (0.9)	Frustrated	2.6 (1.3)
	Curious	3.7 (1.1)	Disappointed	2.2 (1.3)
	Optimistic	3.1 (1.1)	Confused	1.6 (0.8)
	Happy	2.9 (1.1)	Disgust	1.6 (0.9)
	Proud	2.7 (1.2)	Bored	1.5 (0.7)

**Table 3:** Descriptive statistics of emotional state.

	Before Task	After Task
Variables ^1^	*M* (*SD*)	*M* (*SD*)
Positive activation	2 (0.4)	2.4 (0.5)
Positive deactivation	1.9 (0.5)	2.0 (0.6)
Negative activation	2.1 (0.3)	2.0 (0.4)
Negative deactivation	1.5 (0.3)	1.9 (0.3)

^1^ Emotional variables are on a five-point Likert scale.

**Table 4:** Descriptive statistics of emotional state and performance by experimental groups.

	SEB		NSEB		CG	
Variables ^1^	*M* (*SD*)	*Mdn*	*M* (*SD*)	*Mdn*	*M* (*SD*)	*Mdn*
Positive activation	2.1 (0.3)	2	2.1 (0.5)	2	1.8 (0.4)	1.8
Positive deactivation	2.0 (0.7)	2	2.1 (0.4)	2	1.6 (0.4)	1.5
Negative activation	2.1 (0.3)	2	2.05 (0.4)	2.1	2.1 (0.3)	2.1
Negative deactivation	1.4 (0.3)	1.5	1.5 (0.3)	1.5	1.5 (0.3)	1.5
Needle holder	49 (7.9)	52.1	55 (17.7)	51.9	49.5 (3.6)	50.8
Clamp	51.4 (2.4)	51.5	49.1 (3.1)	50.1	47.5 (3.9)	48.4
Suture	9.4 (2.3)	10	10.1 (2.3)	10.5	10.9 (2.3)	12

^1^ Emotional variables are on a five-point Likert scale. The clamp and needle holder scores are interpreted in rotation angle. “SEB” = Self-Efficacy Belief group; “NSEB” = No Self-Efficacy Belief group; “CG” = Control Group.

**Figure A1:**
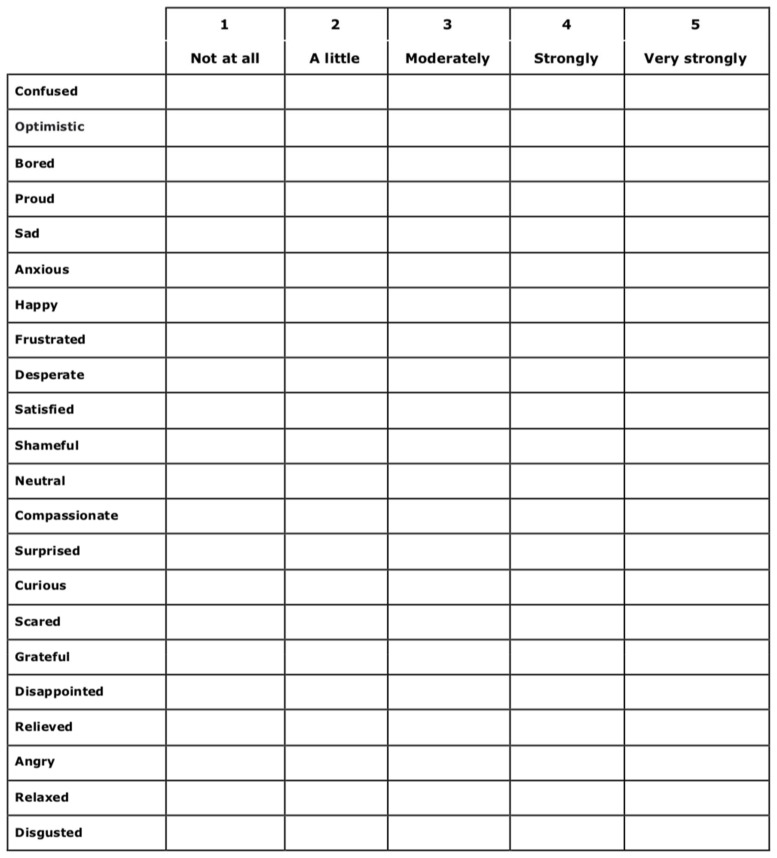
Self-reported questionnaire.

## Appendix A. Self-Efficacy Belief and Task Instructions (SEB Group)

You will have to perform a terminal anastomosis suture task on a pelvic trainer using laparoscopic instruments (a forceps and a needle holder). You will be fitted with the mixed reality headset by the examiner and the robotics center team and your actions will be assessed using the motion capture system. You will be asked to complete the suturing task within a time limit of 20 min. Before and at the end of each session you will be asked to fill in a self-evaluation questionnaire. Remember that the study is anonymous, so you will have to indicate your participation number (not your full name), your age, and your year of study. You will have a total of three sessions to master the suture procedure.

This task is new to you and may cause you difficulties, so it is up to you to find ways to overcome them. It is normal to have difficulties with a new task, so the aim is to overcome them in order to be as successful as possible in the suturing task you are asked to perform. If you are able to perform this task, you will have five sessions to master the suturing gesture.

If you have difficulties, or the material seems complex or impressive, remember that it is a way of improving your skills and concentrate on the suture. Even if the method differs from what you may have learned before, laparoscopic suturing tends towards the same goal, the major difference is that mastering this learning will allow you to perform your operations faster, and with less physical after-effects (scar type) for your patients.

Feedback will be given to you during each session via the mixed reality headset.

Wishing you good luck with the experimentation.

And remember: Stay focused, you can do it, you are capable of it.

Poitiers ABS Lab CLLE Toulouse

## Appendix B. Self-Reported Questionnaire (Medical Emotion Scale) Given to the Participants before and after the Task

Using the scale below, indicate how you are currently feeling, (bearing in mind the activity you will/you have just completed. For each emotion, please indicate the strength of that emotion by selecting the number (from “1- Not at all” to “5- Very strongly”) that best describes the intensity of your emotion.

## Appendix C. Template of the Feedbacks/Disruptive Events via HoloLens-2 for SEB and NSEB Groups

SESSION 1:

The participants arrive in the room after having filled in the documents (consent, starting instructions, self-assessment questionnaire of the emotional state) with the student in charge of the project. The team places them in front of the pelvic trainer, puts on the HoloLens-2. When the team is ready, the audio started and the test begins:

Start: “You may now begin the suturing task”.

10 min: “Are you sure that you are correctly doing the task?”

15 min: “Place your hands correctly!”

16 min: VIDEO FEEDBACK

20 min: “The session is now over”.

The participants leave the room, the student in charge of the project gave them the emotional state self-assessment questionnaire one last time.

SESSION 2:

The participants arrive in the room after having completed the documents (consent, exit instructions, self-assessment of emotional state questionnaire) with the student in charge of the project. The team places them in front of the pelvic trainer, puts the HoloLens-2 on him/her. When the team is ready, the audio started and the test begins:

Start: “You may now begin the suturing task”.

5 min: “What you are doing doesn’t seem right”.

9 min: *Course question*.

10 min: “Place your hands correctly!

11 min: VIDEO FEEDBACK

15 min: “You are doing the task correctly”

20 min: “The session is now over”

The participants leave the room, the student in charge of the project gave them the emotional state self-assessment questionnaire one last time.

SESSION 3:

The participants arrive in the room after completing the documents (consent, exit instructions, emotional state self-assessment questionnaire) with the student in charge of the project. The team places them in front of the pelvic trainer, puts on the HoloLens-2. When the team is ready, the audio started and the test begins:

Start: “You can now start the suture task”.

3 min: “Disruptive and loud Beep”

5 min: “You have successfully completed the task”.

8 min: “Disruptive and loud Beep”

10 min: “Concentrate!”

12 min: “Disruptive and loud Beep”

13 min: “Are you satisfied with your work?”

14 min: VIDEO FEEDBACK

15 min: “Do you want to start all over again?”

17 min: “Disruptive and loud Beep”

20 min: “The session is now over”.

The participants leave the room, the student in charge of the project gave them the self-assessment questionnaire of the emotional state one last time.
